# Low acceptance of osteoanabolic therapy with parathyroid hormone in patients with fragility fracture of the pelvis in routine clinical practice: a retrospective observational cohort study

**DOI:** 10.1007/s00402-019-03241-4

**Published:** 2019-07-22

**Authors:** Norbert Suhm, Alexander Egger, Christoph Zech, Henrik Eckhardt, Mario Morgenstern, Simon Gratza

**Affiliations:** 1 Department of Orthopaedic and Trauma Surgery, University Hospital Basel, Spitalstrasse 21, 4031 Basel, Switzerland; 2 Department of Interventional Radiology, University Hospital Basel, Petersgraben 4, 4031 Basel, Switzerland

**Keywords:** Fragility fracture of the pelvis, Osteoporosis, Osteoanabolic therapy, Teriparatide

## Abstract

**Introduction:**

A recent randomized controlled trial has reported full patient compliance and no adverse events from therapy with parathyroid hormone (PTH) for osteoporosis and accelerated healing of fragility fractures of the pelvis. The purpose of the presented study was to evaluate if similar results can be achieved with comprehensive PTH therapy in routine clinical practice. We hypothesised that patients’ burden of PTH therapy is underestimated in the literature.

**Patients and methods:**

Osteoanabolic PTH therapy was recommended to 79 patients suffering from an acute fragility fracture of the pelvis (FFP). Case finding, initiation of therapy and follow-up were performed by a fracture liaison service team. Primary outcome was PTH initiation rate. Secondary outcomes were implementation rate of alternative antiresorptive pharmaceutical therapy for osteoporosis and participation rate in a bone metabolic workup. Adverse events and effects potentially related to the therapy with bone-active drugs were documented as exploratory outcomes.

**Results:**

Osteoanabolic PTH therapy as suggested was accepted by 32%, whereas antiresorptive therapy was implemented in another 14% of the patients. DEXA scans were available in 38% of the patients (+ 27% when compared to baseline). A bone-specific laboratory analysis was done in 18 patients, uncovering 7 pathological findings. Two patients terminated PTH therapy early because of side effects.

**Conclusion:**

The experiences with PTH therapy in FFP patients with respect to, implementation rate, frequency of side effects and of pathological findings in laboratory controls as reported from a previous RCT could not be reproduced in routine clinical practice.

## Introduction

With the demographic change, the number of patients with a fragility fracture of the pelvis (FFP), concomitant osteoporosis but also high functional demand steadily increased [1]. Rommens and Hofmann [2] developed a dedicated classification of FFP also providing a recommendation with respect to operative or non-operative treatment of the acute fracture. Irrespective of this decision, measures for secondary fracture prevention according to the specific needs of these patients must be considered too.

The first consultation of a patient who has recently suffered from a fragility fracture with the orthopaedic surgeon presumably represents a unique opportunity not only for treating the fracture, but also for initiating diagnostic measures and osteoporosis therapy. However, despite this setting and clinical guidelines, the literature has only reported a low percentage of patients with FFP receiving bone-active drugs following orthopaedic surgery [3, 4]. While several factors may contribute to the low proportion of patients offered medical treatment for osteoporosis directly after a fracture, one reason may be uncertainty among orthopaedic surgeons regarding potentially negative interference between these medications and fracture healing [5, 6].

Currently, the two types of bone-active drugs are antiresorptive and osteoanabolic medications, although antiresorptive medications such as bisphosphonates are most commonly used [4, 7]. However, concerns regarding potential negative effects of these medications on fracture healing have been raised [5]. Moreover, a human monoclonal antibody to receptor activator of NF-κB ligand (Prolia® or denosumab) has been developed as an alternative antiresorptive therapy of osteoporosis, yet to date there are no published reports on the effect of denosumab on fracture healing in humans.

In contrast, there are reports on controlled trials in humans [8–11] about the off-label use of parathyroid hormone (PTH) for accelerating fracture healing. From an orthopaedic surgeon’s perspective, this additional effect of anabolic compounds on fracture healing seem more favourable for use in osteoanabolic therapy of osteoporosis compared with antiresorptive drugs. This tendency has been confirmed by several case series and case reports [12–22] despite the little evidence and a potential lack of reimbursement.

Peichl et al. [10] reported data from a randomized control trial (RCT) that evaluated osteoanabolic PTH therapy for its combined effect on osteoporosis and on fracture healing in elderly patients who were suffering from FFP. Significantly shorter time to fracture healing and improved functional outcome were found in the therapy arm. However, PTH therapy requires daily subcutaneous application of the drug and regular laboratory checks. Nevertheless, Peichl et al. [10] reported that PTH therapy achieved full patient’s compliance with no complications. However, it has been previously noted [23] that real-world post-fracture care differs from care delivered in an RCT.

The purpose of our study was to test the hypothesis that the data reported by Peichl et al. [10] underestimate patient’s burden of PTH therapy. Therefore, we evaluated whether the experience with comprehensive PTH therapy as reported in the RCT by Peichl et al. can be reproduced in an unselected sample of patients with FFP in routine clinical practice.

## Methods

The analysis was based on medical records as well as on a follow-up examination in an observational cohort of 79 patients suffering from an FFP. The analysis was part of internal quality assurance measures and conducted in accordance with Swiss laws, good clinical practice, following the CIOMS Criteria and the Declaration of Helsinki after approval by the local ethical committee (Ethics Commission Northwest Switzerland, EKNZ 2015-299).

### Patients

Patients presenting with an FFP at the emergency department of a trauma centre covering an overaged population of > 300,000 citizens were retrospectively identified. Patients had been admitted to the hospital for pain in the pelvic area after minimal or no trauma. The diagnosis of FFP was based on reported low-energy mechanism of injury combined with radiologic confirmation of a pelvic fracture.

As in all fragility fractures, two major aspects have to be considered: the type of treatment of the specific fracture (operative or non-operative) and secondary fracture prevention. The facts contributing to the decisions for operative vs. non-operative treatment of FFP and the results of the respective treatment are presented elsewhere [24]. In theory, there are five possibilities regarding therapy using the two categories of bone-active drugs available for secondary fracture prevention (Table 1):

- *“Osteoanabolic” group*: patients who received once-daily subcutaneous injection of 20 μg of recombinant teriparatide and laboratory analysis of bone metabolism according to schedule.
- *“Antiresorptive” group*: patients with contraindications against teriparatide application (e.g., severe renal dysfunction or radiation therapy involving bony structures) or patients who declined osteoanabolic therapy for other reasons were offered antiresorptive therapy of osteoporosis if indicated according to national guidelines [25].
- *“Switch” group*: in all patients who already received antiresorptive drug therapy at the time when sustaining a FFP, a switch to osteoanabolic teriparatide therapy was suggested.
- *“None” group*: patients without indication for specific therapy for osteoporosis or not willing to accept any type of such therapy in spite of an indication.

**Table 1:** Diagnostics of osteoporosis done prior to or after suffering from FFP per type of implemented therapy with bone-active drugs

	Type of therapy			
	Osteoanabolic	Antiresorptive	Switch	None
Prior to FFP				
* n*		7		72
F DEXA		3		6
Indication for Tx		1		24
After FFP				
*n*	9	7	7	27
F DEXA	3	4	2	2
Lab	7	2	3	6
Indication for Tx	6	5	4	20

Nine patients shifted from the “None” group prior to FFP to the “Osteoanabolic” group after FFP and another seven patients shifted from the “None” group prior to FFP to the “Antiresorptive” group after FFP. All patients in the “Antiresortive” group prior to FFP switched to osteoanabolic therapy: occupation of the “Switch” group after FFP

*n* number of patients, *F DEXA* number of DEXA scans performed, *Lab* number of patients in whom results from bone specific laboratory analyses were available, *Indication for (osteoporosis) Tx* according to national guidelines

**Fig. 1:**
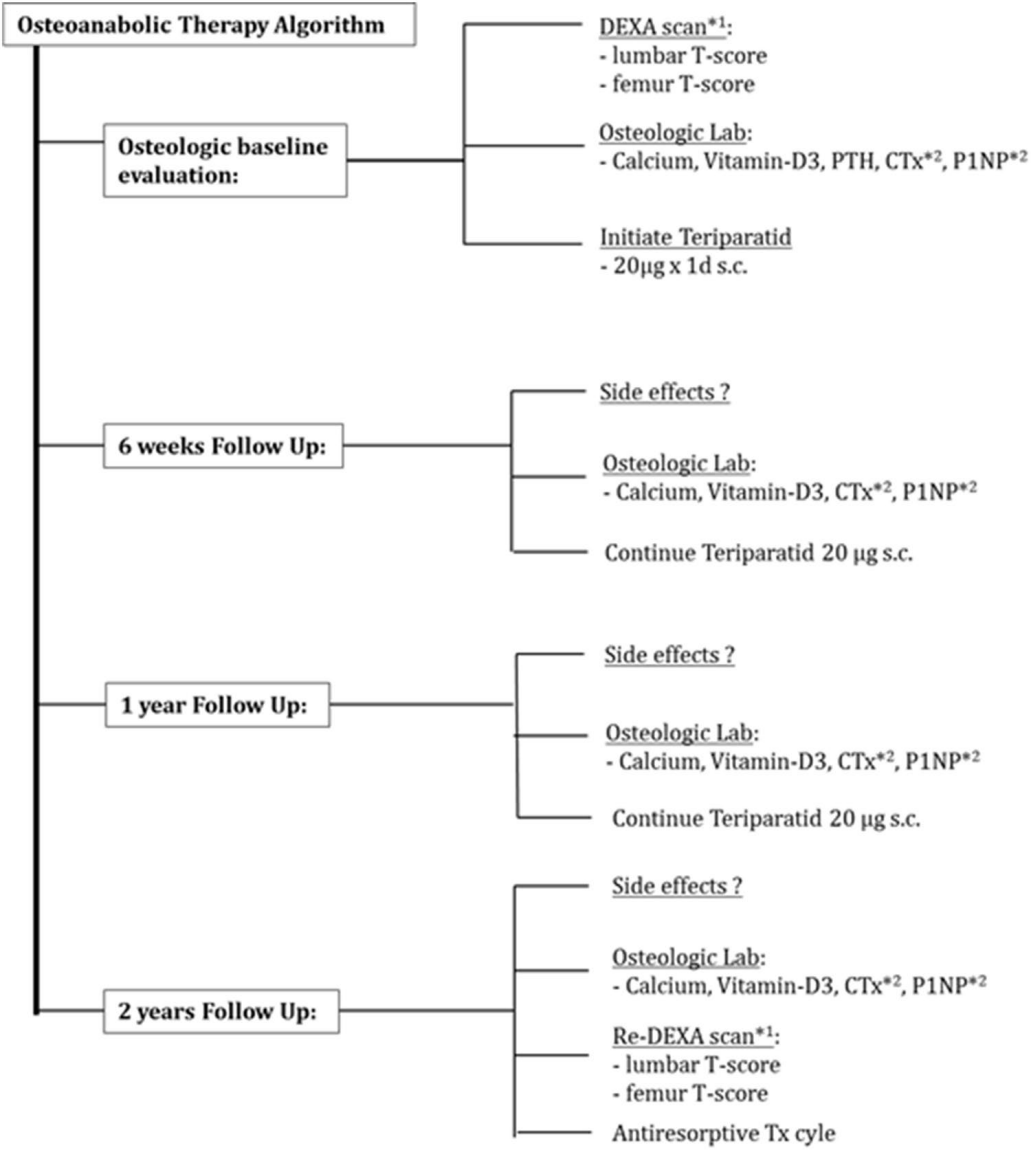
Timing and type of diagnostic measures proposed to all FFP patients receiving osteoanabolic teriparatide therapy. The osteoanabolic therapy cycle should be finished with transition to an antiresorptive medication to prevent non-mineralized osteoid from resorption. DEXA^*1^ double energy X-ray absorptiometry (done only if last DEXA was 2 years or older), *T-score* the measurement unit to report quantitative results from DEXA scan, *PTH* parathyroid hormone, the natural substance, CTx^*2^ C-terminal cross-linking telopeptide, a bone (resorption) turnover marker, P1NP^*2^ procollagen type I N-terminal propeptide, a bone (formation) turnover marker

Because no patient received osteoanabolic therapy prior to the FFP, there was no “Inverse Switch” group of patients switching from osteoanabolic to antiresorptive therapy. All patients in the “Osteoanabolic” group and in the “Switch” group were invited for dedicated follow-up visits comprising specific blood tests and assessment for adverse reactions (according to Fig. 1).

All patients had been evaluated according to the criteria below:

#### Inclusion criteria:

- Elderly patients (age, *z* ≥ 65 years) of any ethnic/sociodemographic background
- Pelvic fracture after low-energy trauma or without any trauma

#### Exclusion criteria:

- Suspicion of a pathological fracture in the context of known or unknown malignancy
- Contraindication against PTH therapy
- Pelvic fracture due to high-energy trauma

All identified patients were contacted and invited for a dedicated clinical and radiological follow-up in 2016, which was at a minimum of 1 year after diagnosis of FFP.

### Data collection

In our institution, a Fracture Liaison Service (FLS) assures implementation of measures for secondary fracture prevention in all patients suffering from fragility fractures of any anatomical site. The workup for osteoporosis specifically adapted to patients with FFP patients comprised the following:

- DEXA (= dual energy X-ray absorptiometry) scan if none had been obtained prior to the fracture or if the most recent DEXA scan was more than 2 years old.
- Review of routine laboratory analyses focusing on bone metabolism and nutrition (e.g., albumin, calcium, creatinine, renal filtration rate).
- Risk–benefit analysis with respect to pharmaceutical therapy of osteoporosis and evaluation of potential contraindications against osteoanabolic or antiresorptive therapy of osteoporosis. As an exemption, osteoanabolic teriparatide was suggested in the first instance to all eligible patients with FFP given the positive effects on fracture healing.

All the captured data are listed in Table 2.

**Table 2:** Data captured to describe patient’s demographics, and to analyse main or additional outcomes

Baseline demographic data
Age
Gender
Patient survival
Data describing population and course of treatment
Patients with prior fractures
Prior diagnostics and therapy for osteoporosis
Confirmation of non-union
Need and reason for reoperation
Risk factors for osteoporosis and non-union
Assessment of main outcomes
Number of patients with type of therapy implemented
Number of patients with diagnostics of osteoporosis performed
Compliance with dedicated protocol: number of patients with bone specific laboratory follow-up
Assessment of additional outcomes
Incidence of repetitive fractures
Number of adverse events under bone active drug
Problems with fracture healing
Need for (revision) surgery

### Baseline data collection

Demographic data (age, sex) were retrieved from medical records. Descriptive data included performed diagnostics of osteoporosis and therapy for osteoporosis already implemented prior to the incidence of FFP, as well as the individual risk profile with respect to osteoporosis and non-union as documented during the case finding visit by the FLS team.

### Follow-up data collection

Newly performed diagnostics of osteoporosis and therapy for osteoporosis implemented after the occurrence of FFP were documented in those patients who attended the scheduled follow-up visit in 2016 at our outpatient clinic. New anteroposterior radiographs of the pelvis were obtained and evaluated for signs of non-unions, migration of osteosynthesis material, and other complications. The incidence of new fractures, adverse events possibly related to therapy of osteoporosis, and revision surgery related to the FFP were documented. Patients unable to attend the outpatient clinic were offered a visit at home or were contacted by telephone call either directly or indirectly via caregivers.

### Outcomes

The primary outcome was acceptance or implementation rate of PTH therapy in FFP patients in a real-world setting calculated as the number of patients who were started on this drug divided by the sample size that was available for follow-up.

Those rates identified as secondary outcomes were calculated accordingly and were contrasted by the status of diagnostics and therapy for osteoporosis prior to the FFP in Table 1. The secondary outcomes were: implementation rate of alternative antiresorptive therapy of osteoporosis in FFP patients; rates of participation in a bone metabolic workup, comprising bone mineral testing and laboratory workup. Additional exploratory outcomes were: adverse events possibly related to the use of bone active drugs; manifestation of problems with fracture healing comprising delayed union or non-union; the need for revision surgery in FFP patients; and incidence of new fractures (Table 3).

**Table 3:** Presentation of additional outcomes

	Type of therapy			
	Osteoanabolic	Antiresorptive	Switch	None
*n*	9	7	7	27
Re-fractures	0	0	1	10
Non-union	2	0	3	6
mRF	3	3	4	15
nmRF	13	16	16	89
Revision surgery	2	0	2	6
Non-union	2	–	2	1
Removal of hardware	–	–	–	5

Occurrence of repeat fractures, non-unions as well as load with risk factors for non-unions, the need for revision surgery and the indication thereof

*n* number of patients per type of therapy, *Re-fracture* number of patients with new fracture during observation period in (*n*) patients, *Non-union* number of patients with documented non-union amongst (*n*) patients, *m RF* number of modifiable risk factors for non-union amongst (*n*) patients, *nm RF* number of non-modifiable risk factors for non-union amongst (*n*) patients, *Revision surgery* number of patients who were subject to revision surgery

## Results

### Participants’ demographic data

**Fig. 2:**
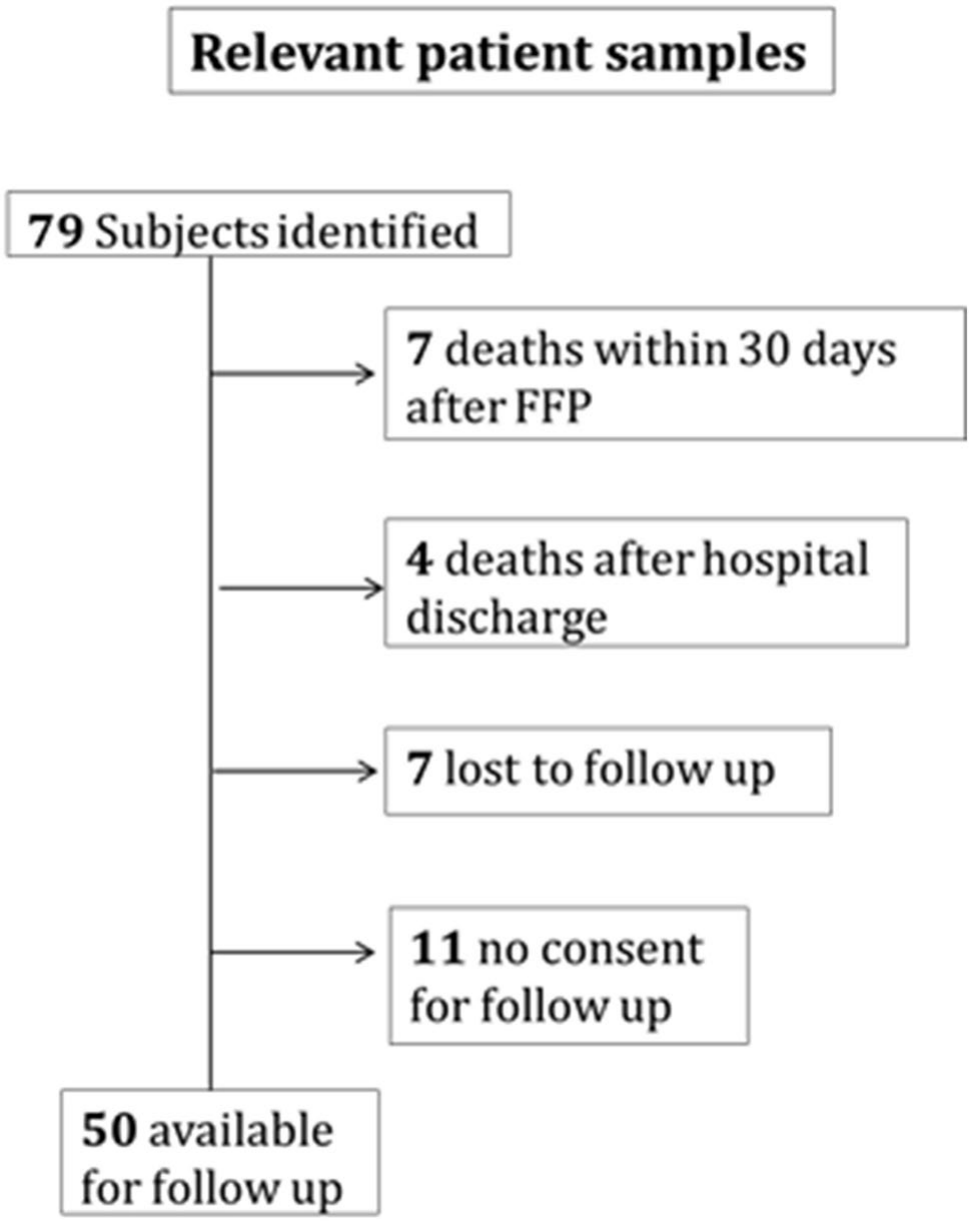
Box chart illustrating the number of patients available for analysis with respect to the relevant outcomes

We identified 79 eligible patients with a mean age of 80.5 years (69 women, 10 men). According to national guidelines, an indication for therapy of osteoporosis was present already prior to FFP in 25 (32%) patients. 30-day mortality was 9% (7 patients), and 13.9% (11 patients) died during the observation period. With 29 patients lost to follow-up or refusal of follow-up investigation, 50 patients could finally be evaluated for all outcomes (Fig. 2).

### Primary and secondary outcomes

#### Primary outcome

All seven patients who were treated with antiresorptive medication prior to the FFP switched to osteoanabolic therapy. Another nine patients agreed to start osteoanabolic therapy with teriparatide.

#### Secondary outcome

Seven patients decided upon an antiresorptive medication. Overall, in 23 of 50 patients (46%), therapy with bone-active drugs was finally implemented. DEXA scans were newly performed in 11 patients after suffering from the FFP. Results from DEXA scans could therefore be evaluated in 19 of the 50 patients (38%), as one DEXA scan was done as a repeat scan. Dedicated osteological laboratory serum analyses were done in 18 patients, 10 of which were done in the course of osteoanabolic therapy with teriparatide. Vitamin D values were out of range in three patients, and calcium level was out of range in four patients.

#### Additional outcomes

Concerning adverse reactions, osteoanabolic teriparatide therapy at standard 20 μg/day was well tolerated by all but two patients. These two patients terminated teriparatide therapy prematurely due to mild adverse reactions (headache, hypertension). An association to the novel therapy has been interpreted as possible. The further course, especially the regression of the reported symptoms, could not be clarified. No adverse reactions were reported by patients taking antiresorptive medication.

Non-unions were observed in 11 of the 50 patients who were available for the follow-up (22%). Five patients (10%) had revision surgery for non-union, and five patients (10%) had revision surgery for implant removal (Table 3).

Within the observation period, 15 new fractures occurred in 11 patients (2 patients with 3 concurrent fractures each) as reported in Table 3.

## Discussion

The most important finding of our study is that patient’s acceptance of and experience with comprehensive PTH therapy carried out as reported in an RCT by Peichl et al. [10] could not be reproduced in an unselected sample of patients with FFP patients in routine clinical practice. This fact is remarkable especially because therapy implementation and post-fracture care were supported by the local FLS team. Such a setup is commonly considered as an established and proven method to achieve recommended standards of care for fragility fractures [26–29].

### Why was this study performed?

PTH-related medications increase the cancellous bone mass and reduce the risk for vertebral and non-vertebral fractures [30] mainly by increasing the longevity of osteoblasts. If PTH could make these cells work harder and longer, more patients might reach functional restoration after a fracture at an earlier time point [31]. Therefore, the off-label use of PTH in patients after FFP may be worth exploring.

A series of animal experiments has confirmed the positive effects of PTH on fracture healing in different species, locations, and under various pathological conditions [31, 32]. However, to date, only four controlled studies of PTH and fracture healing have been carried out in humans [8–11, 33]. Overall, the results of these studies were inconclusive. To define the future role for PTH as an agent for accelerated fracture healing, further clinical studies in various fracture types and patient samples would be needed [6]. However, a double-blind, placebo-controlled study of teriparatide for accelerating fracture healing in femoral neck fractures in men and post-menopausal women had to be terminated early because of low enrolment [34]. This result shows that studies on fracture healing in an aged population are difficult to perform. While our study was not designed to contribute to the little evidence with respect to the effect of PTH on fracture healing, we focused on answering the question of whether there is a real need to initiate these studies on fracture healing in various fracture types as outlined above given that they are difficult to perform. To answer this question, data on patient’s acceptance and experience with comprehensive PTH therapy in routine clinical practice were needed, which—to the best of our knowledge—was not available.

### How do our results compare with data about the RCT by Peichl?

Peichl et al. [10] reported that PTH was implemented in all patients and that all patients participated in a bone metabolic workup representing 100% rate. We can only explain these extremely high rates by the specific study setting in their RCT and would not expect to achieve similar rates in routine clinical practice despite the professional support, for instance, by a FLS. Interestingly, the PTH implementation rate in our study increased when compared to the share of osteoanabolic therapy as reported from its label application [35]. Clearly, there is a need for further clarification regarding the reported 0% rate of pathological lab findings while on therapy, adverse events or patients with early termination of medication application which were also reported by Peichl et al. [10].

### What was the impact of FLS on the study results?

There is extensive literature reporting FLS impact on secondary fracture prevention in general by means of key clinical outcomes including bone mineral densitometry testing, bone health referrals, and pharmacotherapy initiation [7]. Because the goal of our study was not to measure FLS team’s performance, we did not document all eligible outcomes mentioned above but rather focused on the rate of bone mineral testing and rates of pharmacotherapy initiation that can be considered as benchmark parameters for assessing the success of FLS teams in other healthcare systems. In our study, these parameters were only assessed to judge the impact of the FLS team’s support in implementing PTH therapy.

Rates of post-fracture bone mineral densitometry in inpatients and outpatients with a fragility fracture seen in a coordinator-based fracture liaison service were reported to be as high as 84% for inpatients and 85% for outpatients. Of the patients who attended their appointments, 73% of inpatients and 52% of outpatients received a prescription of anti-osteoporosis medication [26] in a very advanced FLS program.

Previous FLS studies had recruitment rates for eligible patients ranging from 28.4 to 61.2% and refusal rates between 0.7 and 61.2% [28]. A more recent meta-analysis also reported that—compared with patients receiving usual care—patients receiving care from an FLS program had higher rates of bone mineral density testing (48.0% vs 23.5%) and treatment initiation (38.0% vs 17.2%) [36]. Considering these outcome parameters, the performance of our FLS team may be rated “intermediate” especially when focusing on the overall therapy implementation rate.

### What are the possible explanations for these findings?

With respect to the primary outcome, we attribute low PTH implementation rate to the patient’s burden related with PTH therapy. This is especially true for elderly patients awaiting transfer to a rehab setting and using a lot of pain medication. These factors are arguments against PTH implementation in the acute fracture setting.

With respect to the secondary outcomes, several factors influenced the key outcome parameters of FLS performance. For instance, the anatomical site of the investigated fragility fracture plays a role. Amongst 1270 patients who entered our FLS program between January 2014 and December 2015 [37], only 6% of patients had a FFP compared with 48% presenting with hip fracture. This observation does not seem to be specific to our geographic region but rather the high number of patient recruitment with hip fracture compared to the low numbers of patients with pelvic fractures that can be found throughout the existing literature on post-fracture care of any type. In a study investigating the effect of a coordinator-based screening program for improving osteoporosis management following fragility fractures comprising a total of 147,071 patients, only 5216 patients had a pelvic fracture (3.5%) compared to 61,102 patients or 41.5% with hip fractures [38]. Similarly, a previous multicentre nationwide investigation [39] on secondary fracture prevention in Switzerland included 3667 fragility fracture patients: 4.1% females and 3.8% males suffered from pelvic and sacral fractures, whereas 22.5% females and 22.3% males presented with proximal femoral fractures. Hence, with respect to our primary outcome—that is PTH implementation rate—FFP may not be the easiest fracture model.

### What can be concluded with respect to quality improvement in fragility fracture care?

Contrary to our consideration mentioned above, we found that all patients with implemented PTH therapy were started as inpatients by the FLS team confirming findings of a recent study. Aubry-Rozier et al. [40] reported initiation of osteoporosis therapy in general with FLS management to be more frequent than with general practitioner management and concluded that the focus should be put on initiation of therapy at the time when patients are still in the hospital rather than delegating this task to the general practitioner. Moreover, to achieve higher therapy implementation rates, it may be necessary to approach the patients repetitively.

A situation frequently described by the FLS team when patients were interviewed in the context of follow-up was that the patients did not know if they had received osteoporosis-specific medication. It is well known that reduced familiarity with treatment decreases adherence. In addition to such patient-level barriers against inclusion in any type of post-fracture care program, there are also provider-level barriers with a potential impact on therapy implementation rates [41]. Information on the suggested therapy may have been unclear or incorrect. Addressing these barriers constitutes a great opportunity for further program improvement at our site. The solutions to these problems reside in dissemination of knowledge on osteoporosis by FLS managers to stakeholders [28], such as general practitioners and other caregivers.

## Limitations

Because of the qualitative nature of our study, it would be inappropriate to extrapolate our findings to the outcomes of a larger program. Moreover, the overall compliance with visits and study procedures in our study was low. This is not unexpected in frail, elderly subjects with a severe fracture, and emphasized the difficulties of performing randomized clinical trials involving frequent, cumbersome postoperative assessments in elderly patients with fractures with potentially substantial transportation issues. Because reported data were also collected by questionnaires completed by caregivers or family members, the information captured has to be interpreted with caution. Nonetheless, the results of our study are relevant, because they reflect the real-world setting.

## Conclusion

Patient’s acceptance of osteoanabolic PTH therapy was found to be inferior when compared to an RCT study setting. There seems to be a limited need to initiate difficult to perform studies on the acceleration of fracture healing with PTH therapy in different fracture types given the low acceptance of such a therapy in a routine clinical care setting.
